# When organs collide: A rare cause of gastrointestinal bleeding

**DOI:** 10.1002/jgh3.13105

**Published:** 2024-07-12

**Authors:** Darragh Egan, Rohita Reji, Tim Mitchell

**Affiliations:** ^1^ Department of Gastroenterology Royal Perth Hospital Perth Western Australia Australia

**Keywords:** endoscopy, gastrointestinal bleeding, gastrosplenic fistula

## Abstract

A 72‐year‐old man was referred to our Emergency Department with a 2‐week history of melaena. His medical history was relevant for Atrial Fibrillation and Non‐Hodgkin's Lymphoma (NHL) in remission on most recent PET. Our patient responded to resuscitative management and then went on to have upper gastrointestinal endoscopic evaluation to elucidate the cause of bleeding. As seen in the images, endoscopy showed a gross defect in fundal wall with evidence of extrinsic infiltration by a large vascular mass‐like structure, suspected to be spleen. Computed tomography (CT) abdomen and pelvis confirmed a gastrosplenic fistula as well as new lymphadenopathy. The findings were in keeping with recurrence of NHL. Discussion at multidisciplinary meeting deemed his gastrosplenic fistula unsuitable for surgical repair. He was managed conservatively, had a nasojejunal (NJ) tube inserted for feeding, and clinically improved on the ward. Our patient expressed a preference not to undergo further chemotherapy, having struggled quite significantly with his initial chemotherapy. He was discharged home 23 days following admission. At this stage, his NJ tube was removed and he was tolerating oral diet. He is currently being managed by the Palliative Care team in the community.

## Graphical abstract

A 72‐year‐old man presenting with upper GI bleeding. Investigations revealed a gastrosplenic fistula eroding into his antrum as the cause.

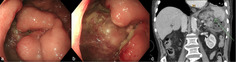

## Case

A 72‐year‐old man was referred to our tertiary hospital with a 2‐week history of melaena. He also described dyspnea and presyncopal symptoms, which were felt to be secondary to anemia. His background history was significant for Atrial Fibrillation, treated with Apixaban 5 mg twice daily and Non‐Hodgkin's Lymphoma (NHL), completed R‐CHOP therapy 1 year prior with complete metabolic remission on Positron Emission Tomography (PET).

Other than anticoagulation with a Factor Xa inhibitor, the patient had no risk factors for gastrointestinal bleeding. He denied any recent steroids, nonsteroidal anti‐inflammatory drugs (NSAIDs) or alcohol intake.

Our patient was pale on presentation and was found to be tachycardic at 110 bpm and hypotensive at 100/70 mmHg. His Hemoglobin was 72 g/L. His abdomen was soft but generally tender.

He was initially managed with fluid resuscitation, intravenous proton pump inhibitor and transfusion of 1 unit of Packed Red Blood Cells. He had an Anti Factor Xa level of 1.62 U/mL and, therefore, received Human Prothrombin Complex (concentrated Factor II, IX, and X).

The patient had appropriate response to this management and his vital signs stabilized. He went on to have upper gastrointestinal endoscopic evaluation to elucidate the cause of bleeding.

**Figure 1:**
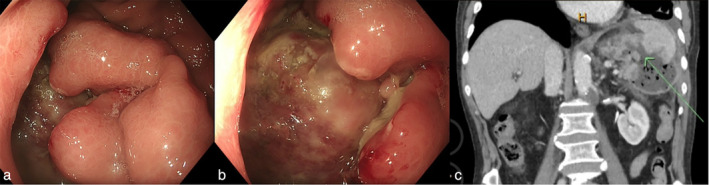
(a,b) Endoscopic images of a gross defect in fundal wall with evidence of extrinsic infiltration by a large vascular mass‐like structure, subsequently proven to be his spleen. (c) Contrast enhanced CT showing gastrosplenic fistula with contained perisplenic collection.

The patient underwent gastroscopy 10 h after emergency department presentation, the most relevant findings being shown in the photograph (Fig. 1a,b). Endoscopy showed a gross defect in fundal wall with evidence of extrinsic infiltration by a large vascular mass‐like structure, suspected to be his spleen. There was no active bleeding seen at time of endoscopy.

Shortly after his gastroscopy, he underwent a computed tomography (CT) abdomen and pelvis (Fig. 1c). This confirmed a gastrosplenic fistula; it also showed evidence of contained perisplenic collection measuring approximately 8 cm in maximal diameter and new para‐aortic nodal mass.

He went on to have a restaging PET which showed widespread disease recurrence above and below the diaphragm. It described the lesion in question as a complex FDG‐avid combination of splenic disease‐related and inflammatory activity.

Our patient was discussed at a multidisciplinary team meeting and his gastrosplenic fistula was deemed unsuitable for surgical repair. He was managed conservatively with intravenous antibiotics, had a nasojejunal (NJ) tube inserted for feeding, and clinically improved on the ward.

His treating hematologist was involved in discussions; however, the patient expressed that he would not undergo further chemotherapy, having struggled quite significantly with his initial chemotherapy.

He was discharged home 23 days following admission. At this stage, his NJ tube was removed and he was tolerating oral food. He had ongoing care from the Palliative Care team in the community.

## Review

Reviewing the literature, gastrosplenic fistula (GSF) is a very rare clinical condition that occurs primarily secondary to gastric or splenic lymphoma. Other causes include gastric or colorectal adenocarcinoma, benign gastric ulcers, splenic abscess and Crohn's disease. A systematic review of GSF was published in 2022^1^ which identified only 46 case reports of GSF across a period spanning Jan 1950 to Sept 2020, and 23 of these cases were secondary to NHL.

The classic presentation of GSF, as first described in 1984, consisted of left upper quadrant pain, fever, and weight loss. These cases are most often identified on imaging performed either as disease follow‐up or for investigation of this nonspecific pain. Presentation with symptoms of upper GI bleed, as was the case with our patient, is rare with only 18% of cases presenting this way. The most consistent physical exam finding is splenomegaly, present in 80% of reported cases.^2^

Contrast enhanced CT is the investigation of choice to diagnose GSF having picked up all cases of GSF in another systematic review published in 2017.^3^ However, when a patient presents with symptoms of upper GI bleeding endoscopy will be the initial investigation of choice. The most common endoscopic finding is of an ulcer in the fundus or the greater curvature of the stomach. It is quite rare to be able to visualize the direct connection between the stomach and the spleen, with this being possible in just 26% of patients identified with GSF.

## Informed consent

Informed written consent was obtained from the patient for the publication of their information and images.
